# Metabonomics Approach to Assessing the Modulatory Effects of Kisspeptin-10 on Liver Injury Induced by Heat Stress in Rats

**DOI:** 10.1038/s41598-017-06017-1

**Published:** 2017-08-01

**Authors:** Yuanlong Hou, Xiaoyan Wang, Jihui Ping, Zhihai Lei, Yingdong Gao, Zhiyu Ma, Cuicui Jia, Zheng Zhang, Xiang Li, Mengmeng Jin, Xiaoliang Li, Chuan Suo, Ying Zhang, Juan Su

**Affiliations:** 1 College of Veterinary Medicine, Nanjing Agriculture University, Nanjing, 210095 China; 2 Ministry of Education Key Laboratory of Systems Biomedicine, Shanghai Center for Systems Biomedicine, and School of Pharmacy, Shanghai Jiao Tong University, Shanghai, 200240 China; 3 Laboratory Medicine, Nanjing First Hospital, Nanjing Medical University, Nanjing, 320100 China

## Abstract

The protective effects of Kisspeptin on heat-induced oxidative stress in rats were investigated by using a combination of biochemical parameters and metabonomics. Metabonomic analyses were performed using gas chromatography/mass spectrometry in conjunction with multivariate and univariate statistical analyses. At the end point of the heat stress experiment, histological observation, ultrastructural analysis and biochemical parameters were measured. Metabonomic analysis of liver tissue revealed that Kisspeptin mainly attenuated the alteration of purine metabolism and fatty acid metabolism pathways. Futhermore, Kisspeptin also increased the levels of GSH, T-AOC as well as SOD activities, and upregulated MDA levels. These results provide important mechanistic insights into the protective effects of Kisspeptin against heat-induced oxidative stress.

## Introduction

Kisspeptin is a 145 amino acid protein, which is cleaved into 54, 14, 13 and 10 amino acids. Its functions primarily bind to G-protein-coupled membrane receptors (GPR54)^1^. Kisspeptin and their receptor GPR54 play a crucial role in the reproductive functions and onset of puberty. In the central nervous system, it is expressed in the hypothalamus and cerebral cortex and hippocampus^2^. In recent years, studies have found that Kisspeptin stimulates the hypothalamic-pituitary-gonadal aixs and promotes the antioxidant system against oxidative damage^3, 4^.

Heat stress can boost the formation of ROS, as similarities exist in the pattern of gene expression of heat-stressed animals and that which arises due to oxidative stress^5^. Reactive oxygen species (ROS), containing hydrogen peroxide, hydroxyl radical and free radicals play an important role in normal physiology, but their overproduction may be a cause of some pathological states. On the other hand, scavenging enzyme systems, including catalase and superoxide dismutase (SOD), are available to protect cells against damage caused by ROS^6^. The balance between oxidation and reduction reactions regulates the homeostasis of the cell. Oxidative stress also inhibits steroid activity which reduced the testosterone synthesis and metabolism^7, 8^.

Lipids are susceptible to oxidation and lipid peroxidation products are potential biomarkers for oxidative stress status *in vivo* and its related stress^9^. Enhanced ROS production in mitochondria during heat stress leads to non-specific modification of lipids, resulting in bioenergetic dysfunction^10^. Hepatocyte membrane are rich in unsaturated fatty acids that are potentially susceptible to oxidative damage by ROS. Hence, increased levels in lipid peroxides and a decline in antioxidant system might be associated with decreased circulating testosterone and gonadotropins. In addition, it is now well documented that peripheral and intracerebroventricular injection of Kisspeptin evokes a stimulatory effect on the levels of testosterone and gonadotropins^11–13^. The effects of kisspeptin-10 on heat-stress-induced lipid peroxidation in liver tissue are currently unknown. Therefore, they have examined possible effects of kisspeptin-10 on methionine-induced lipid peroxidation in brain and testicle tissue of young male rats^4, 14^. Our study is the first attempt at an analysis of the protective effects of kisspeptin-10 through the perspective of metabonomics.

Our recent metabonomic profiling approach revealed that the contents of metabolites, such as amino acid metabolism and fatty acid metabolism pathway, are highly associated with heat stress^15^. Therefore, we hypothesized that the intervention of Kisspeptin in response to heat stress will attenuate the heat-induced variations in metabolites. To verify this hypothesis, we use kissppetin in the heat-induced model by means of metabolite profiling along with classic pharmacological and biochemical indices.

## Results

### Temperature Assessment

After 2 h of heat exposure, scrotal and body surface temperatures of all rats were significantly elevated. The rectal temperature closed to 40 °C when rats suffered from heat stress, as indicated by accelerated respiratory rate, excessive drinking, and irritability.

### Histopathological Result

**Figure 1:**
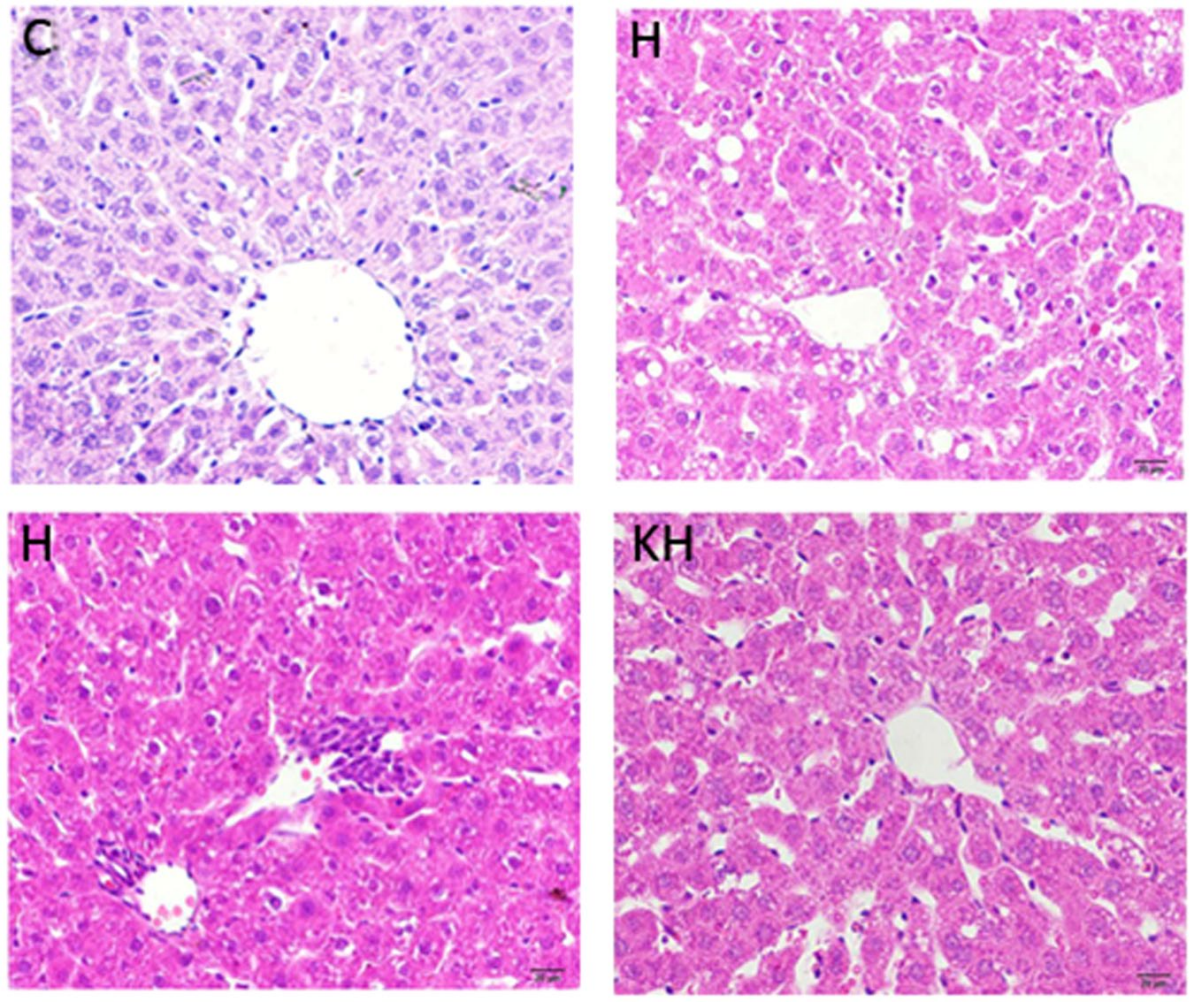
Light microscopy showing normal liver tissue in the control group (HE staining, ×100) (**A**); hepatocyte steatosis in H group (**B**); inflammatory cells and cellular edema in the heat stress group (**C**) and Kisspeptin group (**D**) (HE staining, ×100). C: control group; H: Heat stress group; KH: Kisspeptin+Heat stress group.

Representative photomicrographs exhibiting liver pathology (HE staining) are presented in Fig. 1. Compared with control group, heat stress exposure caused irregular arrangement of the hepatocytes and hepatocyte steatosis. In addition, histological observation also showed that considerable amount of inflammatory cells and cellular edema were found in liver tissues. However, Intervention with Kisspeptin in heat-treated rats remarkably reduced the number of steatotic hepatocytes and there are also no inflammation and apoptosis observed in the KH group. (C: control group; H: Heat stress group; KH: Kisspeptin+ Heat stress group).

**Figure 2:**
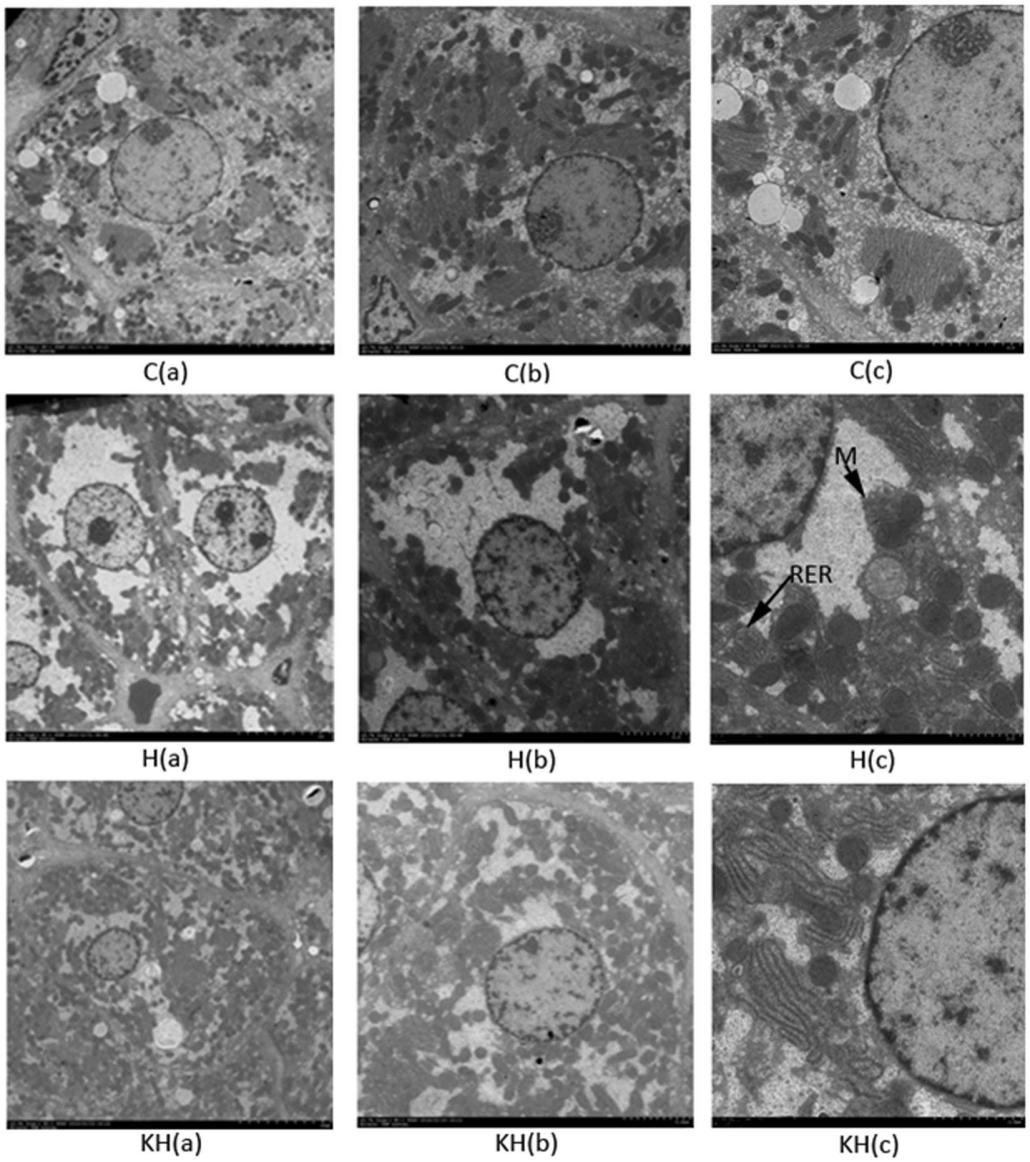
Transmission electron microscopy shows the dissolution of mitochondrial (M) membrane fragmentation (arrows) and the dilation of rough endoplasmic reticulum (RER) (arrows) within liver tissue. (a): Bar = 10 μm; (b): Bar = 5 μm; (c): Bar = 2 μm. C: control group; H: Heat stress group; KH: Kisspeptin+Heat stress group.

Ultrastructural Analysis by Electron Microscopy of the Liver tissue after Heat stress in Rat. Effect of kissppetin observed by transmission electron microscope in heat-treated liver microstructure in Fig. 2. In the H group ((a) ×10 μm, (b) ×5 μm, (c) ×2 μm), the organelle changes included dilation of rough endoplasmic reticulum (RER), mitochondrial (M) membrane fragmentation dissolution were presented by means of transmission electron microscope. In the KH group ((a) ×10 μm, (b) ×5 μm, (c) ×2 μm), there were less rough endoplasmic reticulum to expand, mitochondrial membrane fragmentation to dissolve in hepatocytes.

### Oxidative Stress in Liver Tissue

**Figure 3:**
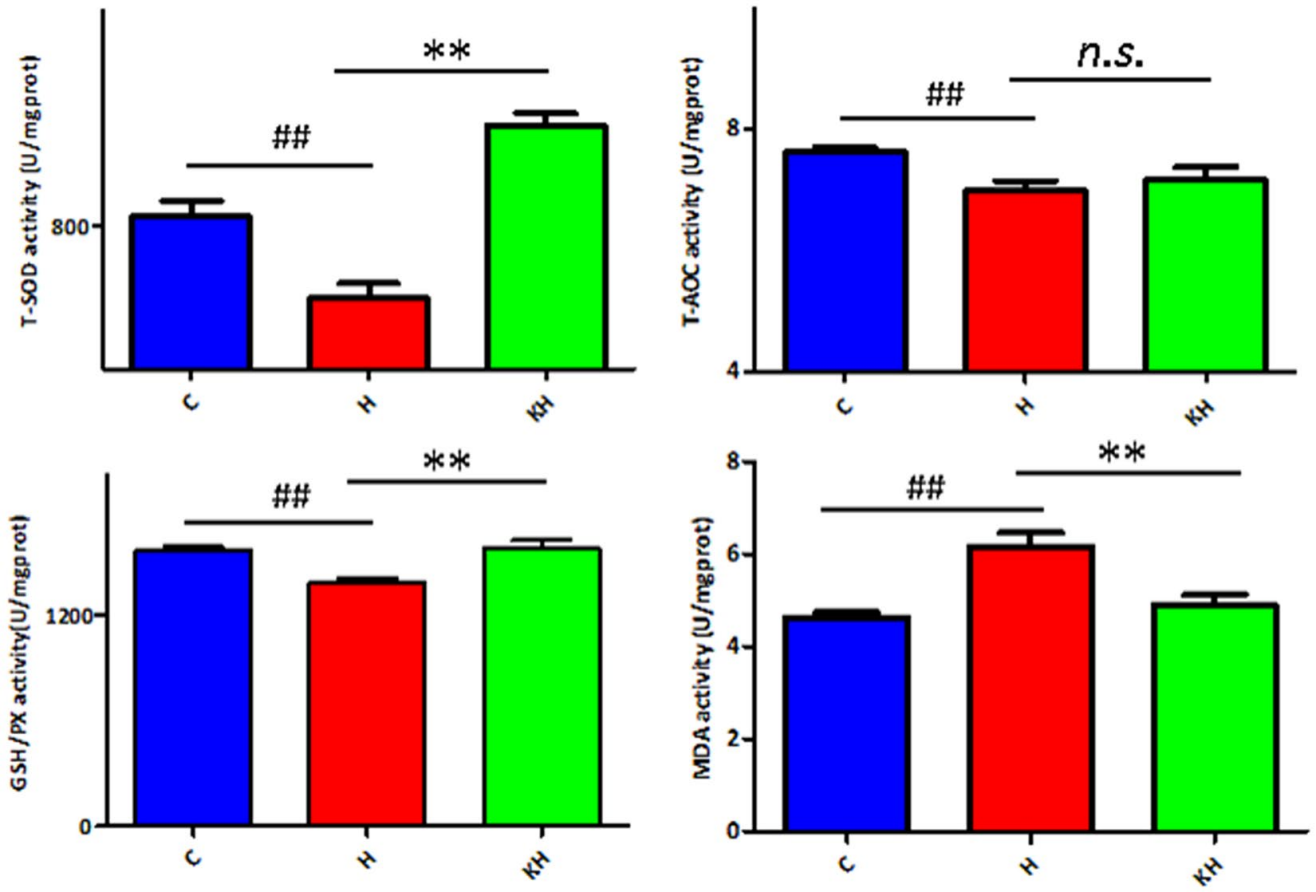
Effects of Kisspeptin on biochemical parameters of liver tissue. Date are expressed as mean ± SD (n = 6). ^##^Indicates p < 0.01 compared to the C group; **Indicates p < 0.01 compared to the H group. C: control group; H: Heat stress group; KH group: Kisspeptin+Heat stress group.

As shown in Fig. 3, the total superoxide dismutase (T-SOD) activity, total antioxidant capacity (T-AOC) and glutathione (GSH) levels significantly decreased in liver tissues of H group rats compared with C group rats (p < 0.01). The malondialdehyde (MDA) contents in the liver tissues of H group rats were significantly higher than C group rats (p < 0.01). However, the intervention of Kisspeptin exhibited protection against heat-induced T-SOD, T-AOC and GSH depletion (p < 0.01 for T-SOD, p < 0.01 for T-AOC, p < 0.01 for GSH, compared with the control group). MDA levels were also lowered by the Kisspeptin intervention significantly (p < 0.01 for MDA, compared with the control group). (C group: control; H group: Heat stress; KH group: Kisspeptin+Heat stress).

### Assessment of serum corticosterone and testosterone level

**Figure 4:**
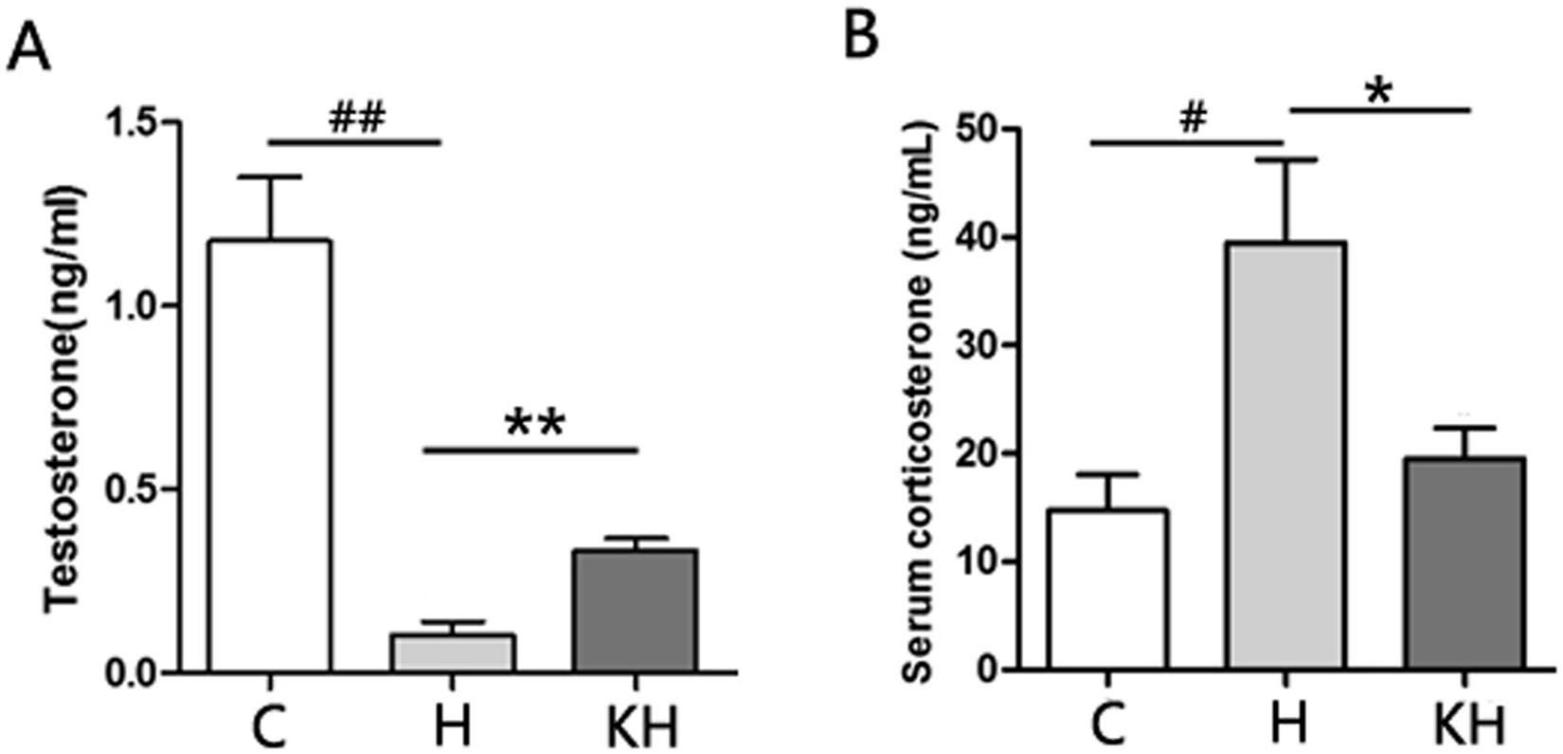
(**A**) Effects of heat challenge and kisspeptin treatment on testosterone level in rat serum. (**B**) Effects of heat challenge and kisspeptin treatment on corticosterone level in rat serum. Date are expressed as mean ± SD (n = 7). ^##^Indicates p < 0.01 compared to the C group; **Indicates p < 0.01 compared to the H group. C: control group; H: Heat stress group; KH group: Kisspeptin+Heat stress group.

Rats exposed to heat stress showed elevated serum level of corticosterone compared to control group, and kisspeptin treatment significantly decreased the corticosterone level compared to heat stressed animals (Fig. 4A). The serum testosterone level was decreased significantly in the H group compared with the C group. The level of testosterone in animals treated with kisspeptin higher than that in the stressed animals, but still lower than that in the control animals (Fig. 4B).

**Figure 5:**
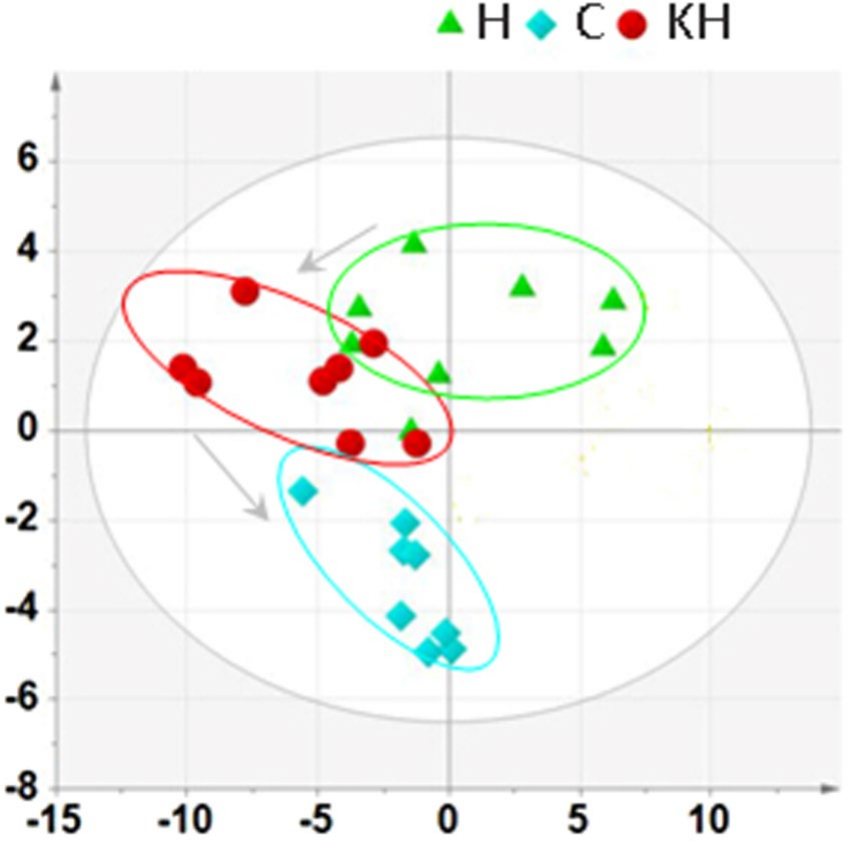
PCA scores plot of all samples in different groups. The blue square is the C group, the red dot is the H group and the green triangle is the KH group. C: control group; H: Heat stress group; KH group: Kisspeptin+Heat stress group.

**Figure 6:**
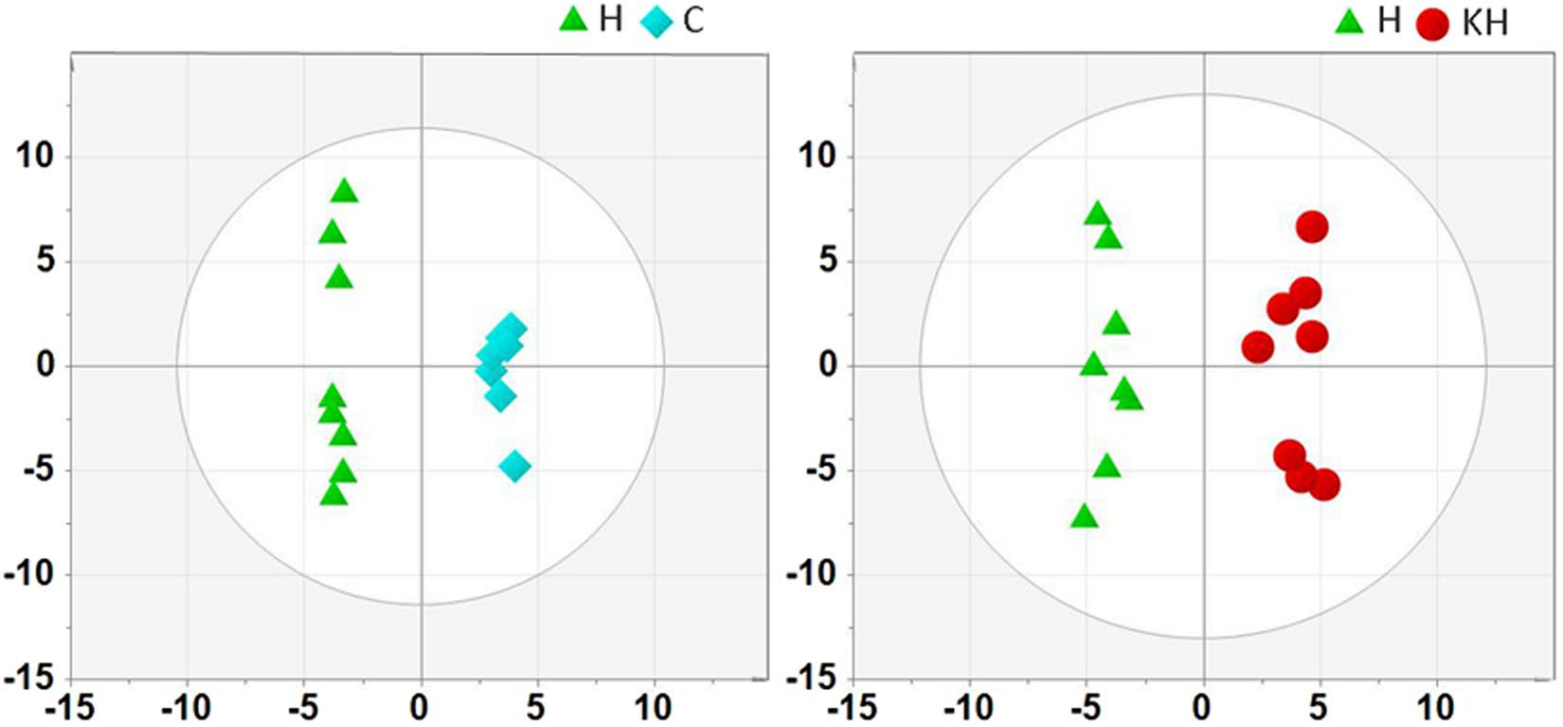
PLS-DA score plots generated from the PLS-DA of the GC/TOFMS data derived from the liver samples of rats. The blue square is the C group, the red dot is the H group and the green triangle is the KH group. C: control group; H: Heat stress group; KH group: Kisspeptin+Heat stress group. (**A**) R2X = 0.644, R2Y = 0.969; Q2 = 0.8; (**B**) R2X = 0.505,R2Y = 0.986,Q2 = 0.798.

Metabolic Variation Induced by Heat Stress. In this study, the scores plot derived from the GC/TOF-MS data in liver sample is depicted in Figure. A clear separation between the H and C groups was observed in the PCA scores plot (R2X = 0.651, Q2 = 0.27) (Fig. 5), and then the scores plot of the PLS-DA model corresponding to PCA model was also shown in Fig. 6. There were liver metabolites identified in different levels between the H and C groups (Fig. 6). Moreover, ANOVA and Kruskal−Wallis tests was used in this metabolite analysis, and the significance threshold was at p = 0.05, and the results of liver sample were shown in Supporting Information Table 1. Fold changes came from the ratio of arithmetic mean values of these metabolites between the H and C group are also shown in this Table 1. A clear separation of metabolic states between the H and C groups was observed, suggesting that exposure to heat stress may lead to metabolic variations. On the other hand, Kisspeptin affected the metabolites variations in different directions (Figs 5 and 6, supplementary Table 2).

## Discussion

To the best of our knowledge, the present study was the first to report that Kisspeptin treatment improved the liver metabolite profile and then relieved liver injury in heat-stress-induced rats. Numerous metabolites, including purine metabolism, lipid metabolism and amino acid metabolism, were changed in the heat stress group and then partially alleviated by intervention of kisspeptin.

Acute and chronic heat stress induce the production of reactive oxygen species (ROS)^5^, deplete cellular antioxidant capacity^16^, and evoke oxidative stress in many tissues, especially in the liver^17^. Abnormal oxidative stress biomarkers, such as MDA, SOD and GSH, are observed in heat stress^18^. MDA is the most abundant product of lipid peroxidation. Previous research had revealed that MDA level was increased significantly in rats exposed to heat stress. SOD is one of the important ROS scavengers, and catalyzes the process of superoxide anion radical (O2′) conversion to hydrogen peroxide (H_2_O_2_). Its activity was inhibited after heat stress. GSH is another ROS detoxicant in the liver. Previous studies demonstrated that hepatic glutathione level was depleted in heat stress rats^19^. Furthermore, there also have been another reports confirming the effects of Kisspeptin treatment on liver oxidant and antioxidant systems^20^. In the present study, heat stress-induced rats showed higher MDA levels, lower GSH level and T-SOD and T-AOC activity in the liver than control rats, whereas Kisspeptin could reverse the above oxidative stress biomarkers (Fig. 3).

The metabolic disturbance induced by heat stress in the rat body may result in disturbance of endocrine system, changes in morphology and dysfunction of some organs. Kiss1/GPR54 are expressed in peripheral tissues including those in the pituitary, gonads, fat, liver^21^ and metabolic status influences Kiss1 expression peripherally. In addition, *in vivo* experiments in mice, both controls and those lacking glucagon receptors in hepatocytes, have revealed that treatment by glucagon stimulates kisspeptin expression^22^. Recent data indicates that the liver as a site of regulated kisspeptin synthesis, define a liver-to-islet endocrine circuit in glucoregulation. As far as we known that stress enhances the activity of the HPA axis and results in increased level of corticosteroids. On the other hand, corticosterone can improve the sensitivity of glucagon and further promote glycogrnolysis. So, in accordance with the level of corticosteroids in our study (Fig. 4), we hypothesize that kisspeptin can reduce the release of corticosterone to suppress glycogrnolysis.

**Figure 7:**
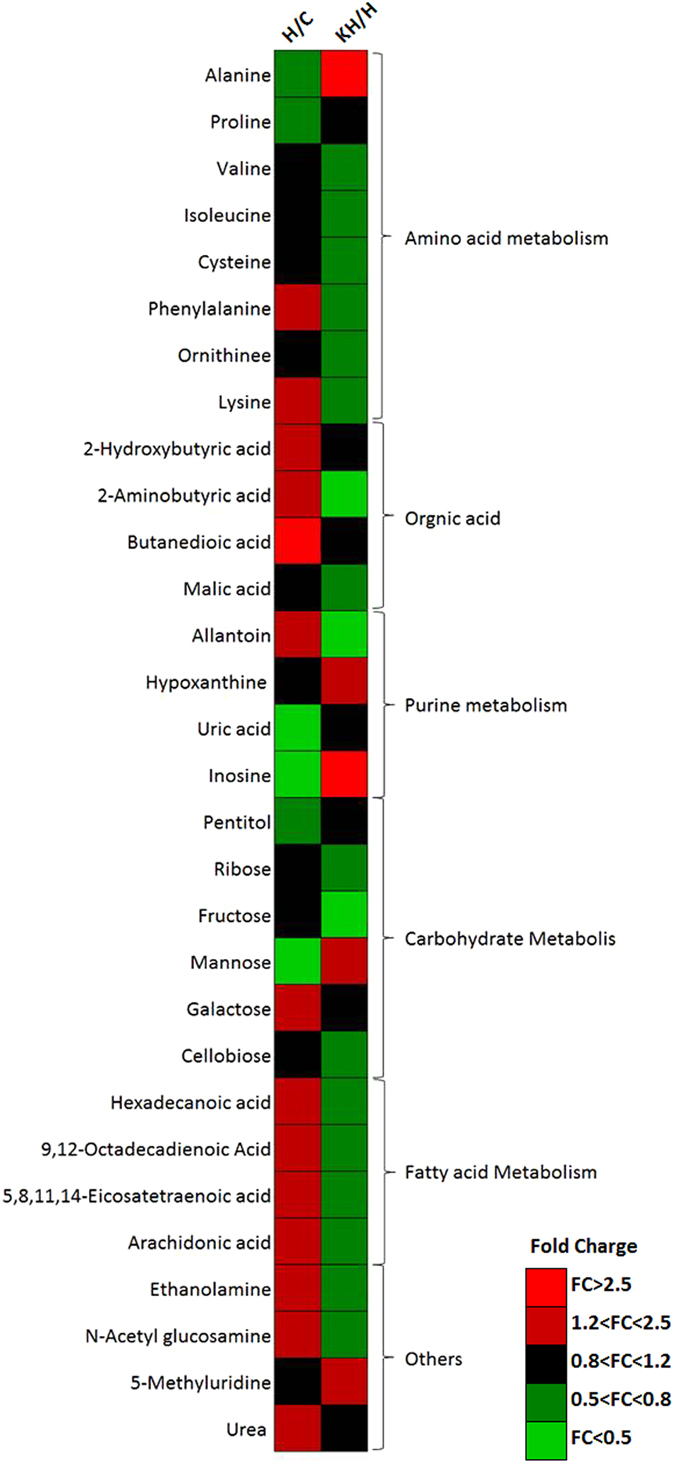
Fold changes of the arithmetic mean values (p < 0.05) of heat-induced changes in liver metabolites by Kisspeptin administration at the study end point. C: control group; H: Heat stress group; KH group: Kisspeptin+Heat stress group.

In our study, the concentrations of free fatty acids (FFAs), such as arachidonic acid, palmitic acid were significantly increased in the heat stress group, which may suggested that heat stress causes oxidative stress and induces apoptosis in the liver (Fig. 7). There is considerable support of the fact that free fatty acids in liver and skeletal muscle cell lines, such as palmitic acid or arachidonic acid, have reported damaging effects, causing reduced cell proliferation^23^, increased reactive oxygen species (ROS) levels^24, 25^, and apoptosis^26^. Moreover, the finding of the current studies also indicated that disrupt the mitochondrial membrane potential^27^ and induce endoplasmic reticulum stress^28^, especially in palmitic acid and arachidonic acid. In addition, the organelle changes were also in agreement with our finding, which included dilation of rough endoplasmic reticulum, mitochondria membrane fragmentation by means of transmission electron microscope (Fig. 2). Notably, Kisspeptin-10 treatment significantly decreased liver free fatty acids (FFAs) including arachidonic acid, palmitic acid and hexadecanoic acid compared with control group. Based on the results above oxidative stress biomarkers, it can be concluded that kisspeptin-10 have the capacity to restore heat-stress-induced liver injury with antioxidant properties in the presence of oxidative damage.

**Figure 8:**
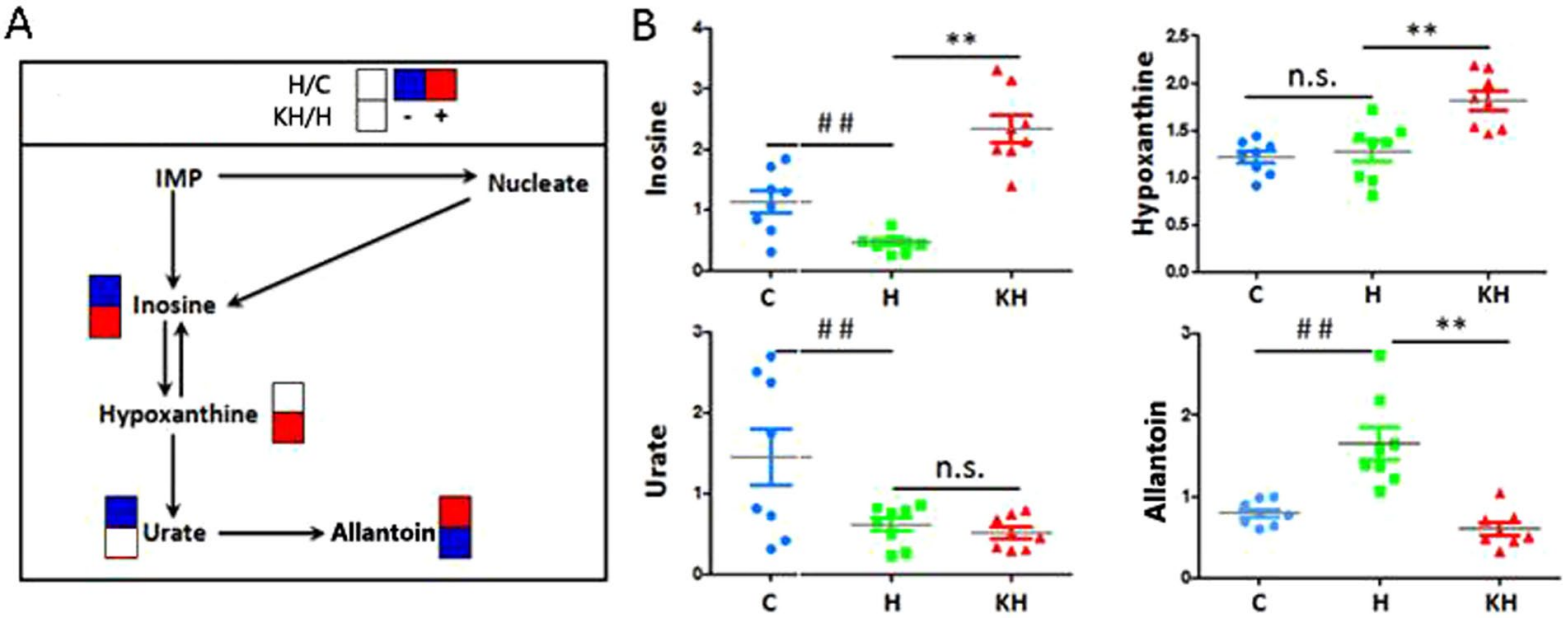
(**A**) The changed purine metabolic pathways in response to heat exposure after Kisspeptin intervention; (**B**) Normalized mean peak areas of metabolites in rats intervened with Kisspeptin compared with the heat stress group.(n = 8). In the figure A, the red square denotes an elevated concentration of metabolites present in the liver tissue, whereas blue square means a reduced level of metabolites. IMP,Inosine 5-monophosphate; Urate,Uric acid. ^##^Indicates p < 0.01 compared to the C group; **Indicates p < 0.01 compared to the H group. C: Control group; H: Heat stress group; KH group: Kisspeptin+Heat stress group.

Under physiological conditions, unlike human, rat can express the enzyme uricase converting the uric acid to allantoin, therefore, allantoin is a final oxidation product of purine metabolism^29^. In our study, higher hepatic metabolism allantoin, and a lower hepatic metabolism of inosine and urate, were also observed in liver samples of animals exposed to heat stress and is indicative of elevated purine metabolism (Figs 7 and 8). As far as we know that both urate and allantoin are derived from purine metabolism, and two key steps of purine metabolism are decided with xanthine oxidase (XO), which catalyzed the hypoxanthine from xanthine to UA, along with the generation of ROS^30, 31^. UA and allantoin had been used as potential markers for monitoring oxidative status in humans^32^. Therefore, the increased UA and allantoin indicated activation of XO and its derived ROS production in the liver of heat-induced rats. On the other hand, compared with the heat stress group, the amounts of metabolites which associated with purine metabolism, such as inosine, hypoxanthine and allantoin, show an opposite trend under the high dose of Kisspeptin-10 treatment group (Figs 7 and 8), which suggested that the high dose of Kisspeptin-10 may be different from the low doses studying the effect on reproductive axis. It may be that the high dose of Kisspeptin-10 play a role in anti-oxidative system, by interfering with the purine metabolism pathway to alleviate heat stress.

Another important finding from the present study was that heat stress affected metabolism and biosynthesis of amino acid in the liver of rats (Fig. 7). Results demonstrated that amino acid metabolism were disturbed by heat stress, leading to lower the levels of phenylalanine and lysine in the liver. Of the two amino acids significantly decreased in heat stress group, phenylalanine, the precursor of catecholamines, is associated with SNS activity, which suggested an up-regulated catecholamine metabolic pathway^15^. Simultaneously, the concentration of lysine in liver was also decreased. Previous literature has addressed that adding zinc supplementation of pigs under heat stress induced changes in amino acid, which included higher level of lysine^33^. Herein, the elevated phenylalanine and lysine levels might play a protective compensation function in response to high temperature. Compared with the heat stress group, our data showed that the concentration of lysine and phenylalanine in the liver were significantly decreased, indicating the protective effects of Kisspeptin on liver damage.

As far as we know that maturity reproductive organ was dependent on the activity of HPG axis and adequate secretion of sexual hormones such as kisspeptin and testosterone. The stress has inhibitory effect on HPG axis through increasing serum corticosterone level^34^. In our study, rats exposed to heat stress showed elevated serum level of corticosterone compared to control group, and kisspeptin treatment significantly decreased the corticosterone level compared to heat stressed animals (Fig. 4B). However, the level of testosterone has a contrary trend comparing with the change of corticosterone (Fig. 4A). So, we deduced that the mechanism kisspeptin may regulated the secretion of gonadotropins by stimulation of upper pathway in Hypophysis Pituitary Gonadal (HPG) axis to alleviate the injury induced by heat stress.

In conclusion, both the classic biochemical parameters and the metabonomic results reveal the protective effects of Kisspeptin in heat-induced rat model. Kisspeptin produced the greatest improvement in GSH, T-AOC and SOD levels compared to heat stress. The hepatic metabonomic results suggest that kissppetin mainly attenuated the alteration of purine metabolism and fatty acid metabolism pathways. Collectively, our results provide important mechanistic insights into the protective effects of Kisspeptin against heat-induced oxidative stress.

## Methods

### Animal Model

Experiments were performed in accordance to Chinese national legislation and local guidelines, and animal experimentations were conducted approved by Nanjing Agriculture University. 24 male SD rats weighing 200 + 20 g (8 weeks old) purchased from Shanghai Laboratory Animal (SLAC, Shanghai, China) were kept a certified standard rat chow and tap water ad libitum (21 ± 1 °C; 45 ± 15%) and controlled lighting conditions (12-h light/dark cycle).

### Experimental Design

The animals were randomly divided into four groups: (C) vehicle (200 μl isotonic saline alone by subcutaneous injection; n = 8) for 7 days, (H) heat stress (40 °C/2 h; n = 8) were exposed to 40 °C for 2 h between 11:00 h and 13:00 h daily for 7 consecutive days with receiving saline by subcutaneous injection, and (KH) kisspeptin-10 (20 nmol/rat/day; n = 8) subcutaneous, starting on day 1 for 7 consecutive days before heat stress. Furthermore, Kisspeptin doses were selected as previously published articles^4, 20^. On the seventh day, all of the animals were deeply anesthetized and decapitated immediately after experiment, and liver sample was collected and stored in liquid nitrogen.

### Biochemical Assay

The T-SOD and activity, GSH and MDA levels in liver tissues were measured with T-SOD, T-AOC, GSH and MDA analysis kits (Nanjing Jiancheng Bioengineering Institute, Nanjing, China) following the manufacturer’s protocols.

### Tissue Processing for Light and Electron Microscopy

The liver tissues were fixed with 10% formalin in 0.01 mol/L of phosphate-buffered saline (PBS) for 3 days. After dehydration, the sample are embedded in paraffin. Then, serial transverse sections at 5 μm were cut, mounted on glass slides, and stained with routine hematoxylin and eosin (HE) for histochemical analysis.

Grafted livers were fixed in 4% glutaraldehyde for 6–8 h at 4 °C, then the sections were mounted on copper grids and stained with uranylacetate and lead citrate and were observed using a Morgagni 268 D transmission electron microscope (FEI Company, Portland Oregon, USA).

Measurement of corticosterone and testosterone level in serum. Corticosterone concentrations detection were carried out by an ELISA kit purchased from R&D Systems (Catalog Number KGE009). The minimum level of corticosterone detection of the kit was 12 pg/mL. Briefly, added 50 μL of Corticosterone Primary Antibody Solution (except the non-specific binding wells, NSB wells) to each well and incubated for 1 hour at room temperature. Each well was rinsed with Wash Buffer before adding samples. 50 μL of standards or serum samples were then added to appropriate wells in duplicate; 50 μL of Calibrator Diluent RD5-43 was added to NSB wells and zero standard (B0) wells. Then, 50 μL of the Corticosterone Conjugate were added to all wells and incubated for 2 h at room temperature on the shaker. After the plate was washed using Wash Buffer for four times, 200 μL of Substrate Solution were added to each well and incubated for 30 min at room temperature. The optical density (O.D.) of corticosterone was read at 450 nm using a microplate reader set within 30 min after the reaction was terminated by adding 100 μL of the Stop Solution. The concentration of corticosterone was calculated according to the standard curves.

Double-antibody 125I-testosterone Radioimmunoassay Kits (Beijing North Institute of Biological Technology, Beijing, China) was applied to assay testosterone level in the serum.

### Sample Pretreatment and GC/TOFM Analysis

The metabolites of organization extraction procedure followed our previous publication. Homogenize Liver tissue as small as 25 mg in the 250 μL mixture of acetonitrile, chloroform, and water (2.5:1:1 v/v/v) for 1 min. The liver homogenate were kept at −20 °C for 20 min, then the mixture was centrifuged at 12 000 g for 10 min. the supernatant get from the mixture was directly put into a new tube. Using the 250 mL of methanol withdraw the residue for the second extraction, followed by centrifugation at 12 000 rpm for another 10 min. The later supernatant was combined with the previous extraction and introduced to a new GC vial and then blow dried under a N2 gas stream. The residue was derived with 50 μL of methoxyamine (15 mg/mL in pyridine) at 30 °C for 90 min and followed by 80 μL of BSTFA (1%TMCS) at 70 °C for 60 min. A 1 μL of solution of derivatives was injected into an Agilent 6890N gas-chromatograph coupled with a Pegasus HT time-of-flight mass spectrometer (GC/TOF, Leco Corp., St. Joseph, MI) at 260 °C in splitless mode. Briefly, a 1-μL derivate was injected with a splitless mode at 270 °C. The flow Metabolite separation was achieved on a DB-5ms capillary column with helium as the carrier gas at a constant flow rate of 1.0 mL/min. The GC temperature programming was set at 80 °C for 2 min, and then ramped to 180 °C with 10 °C/min, to 230 °C with 6 °C/min, finally to 295 °C/min with 40 °C/min and hold for 8 minutes. The transfer interface and ion source was set to 270 °C and 220 °C, respectively. Data were acquired with m/z range of 30 to 600 at an acquisition rate of 20 spectra per second.

### Statistical Analysis

The acquired gas chromatography–time-of-flight mass spectrometry (GC–TOFMS) data were processed (including smoothing, denoising, peak picking, identification, and alignment) using ChromaTOF software (v4.22; Leco Co.) as described in a previous publication. Sample information, peak retention time, and peak area (quant mass) were included in the final dataset. Known artificial peaks, such as peaks caused by noise, column bleed, and N,O-Bis(trimethylsilyl) trifluoroacetamide (BSTFA) derivatization agents, were removed from the dataset. Partial least-squares-discriminant analysis (PLS-DA) validated the principal component analysis (PCA) model were performed with SIMCA-p software to identify the metabolites differentially. The variable importance in the projection (VIP) values (VIP > 1.0) are considered to be differentiating variables. Fold changes of the arithmetic mean values the ratio of among four groups and T-test (P < 0.05) was used for further differentiating variables selection and validation.

## Electronic supplementary material

Supplementary Info 1
