# Supplementary material for: Metabonomics Approach to Assessing the Modulatory Effects of Kisspeptin-10 on Liver Injury Induced by Heat Stress in Rats
Source: Sci Rep. 2017 Aug 1;7:7020. doi: 10.1038/s41598-017-06017-1 (PMC5539146; doi:10.1038/s41598-017-06017-1)
Supplement: Supplementary file 1 — Supplementary Info 1 [file 41598_2017_6017_MOESM1_ESM.doc]

**Metabonomics Approach to Assessing the Modulatory Effects of Kisspeptin-10 on Liver Injury Induced by Heat Stress in Rats**

Yuanlong Hou^1^§, Xiaoyan Wang^2^§, Jihui Ping^1^§, Zhihai Lei^1^, Yingdong Gao^3^, Zhiyu Ma^1^, Cuicui Jia^1^, Zheng Zhang^1^, Xiang Li^1^, Mengmeng Jin^1^, Xiaoliang Li^1^, Chuan Suo^1^, Ying Zhang^1^, Juan Su^1^*

^1^College of Veterinary Medicine, Nanjing Agriculture University, Nanjing 210095, China,^2^Ministry of Education Key Laboratory of Systems Biomedicine, Shanghai Center for Systems Biomedicine, and School of Pharmacy, Shanghai Jiao Tong University, Shanghai 200240, China. ^3^Laboratory Medicine, Nanjing First Hospital, Nanjing Medical University, Nanjing 320100, China.

Table1. Differentially expressed metabolites in liver to be accountable for the separation between KH group and H group

|  | | | Average p values of the ANOVA tests |  |
| --- | --- | --- | --- | --- |
| No. | Retention Time | Metabolites |  | Fold Change |
|  | | | KH v.s. H | KH v.s. H |
| 1 | 6.02 | Alanine | 4.04E-05 | 3.97 |
| 2 | 7.01 | 2-Aminobutyrate | 0.001924 | 0.41 |
| 3 | 7.64 | Valine | 0.028737 | 0.78 |
| 4 | 8.38 | Ethanolamine | 0.000157 | 0.70 |
| 5 | 8.75 | Isoleucine | 0.022442 | 0.79 |
| 6 | 8.82 | Proline | 0.032904 | 0.78 |
| 7 | 11.32 | Malate | 0.039667 | 0.79 |
| 8 | 11.88 | Allantoin | 0.000204 | 0.37 |
| 9 | 12.15 | Creatinine | 0.000174 | 0.72 |
| 10 | 12.25 | Cysteine | 0.012486 | 0.70 |
| 11 | 13.06 | Phenylalanine | 0.01098 | 0.69 |
| 12 | 13.67 | Ribose | 0.015474 | 0.78 |
| 13 | 15.41 | Hypoxanthine | 0.002886 | 1.42 |
| 14 | 15.58 | Ornithine | 0.000836 | 0.72 |
| 15 | 16.32 | Fructose | 7.6E-05 | 0.48 |
| 16 | 16.54 | Mannose | 0.003681 | 2.20 |
| 17 | 17.09 | Lysine | 0.003466 | 0.69 |
| 18 | 17.16 | Hexadecanoate | 0.003292 | 0.57 |
| 19 | 19.21 | N-Acetyl glucosamine | 0.036174 | 0.73 |
| 20 | 19.41 | Myo-inositol | 0.000859 | 0.79 |
| 21 | 19.49 | Octadecadienoate | 0.011473 | 0.54 |
| 22 | 21.33 | Eicosatetraenoate | 0.00248 | 0.55 |
| 23 | 21.98 | Arachidonate | 0.001023 | 0.54 |
| 24 | 22.12 | 5-Methyluridine | 0.000176 | 2.6 |
| 25 | 22.97 | Inosine | 1.06E-06 | 5.1 |
| 26 | 23.39 | Cellobiose | 0.034443 | 0.63 |
| 27 | 23.90 | Maltose | 0.039479 | 0.80 |

Table2. Differentially expressed metabolites in liver to be accountable for the separation between H group and C group

|  | | | Average p values of the ANOVA tests |  |
| --- | --- | --- | --- | --- |
| No. | Retention Time | Metabolites |  | Fold Change |
|  | | | H v.s. C | H v.s. C |
| 1 | 6.33 | 2-Hydroxybutyrate | 0.005807 | 1.71 |
| 2 | 6.51 | Proline | 0.013057 | 0.66 |
| 3 | 7.01 | 2-Aminobutyrate | 0.006887 | 2.10 |
| 4 | 7.12 | Urea | 0.002955 | 1.45 |
| 5 | 8.38 | Ethanolamine | 0.000477 | 1.35 |
| 6 | 9.03 | Butanedioate | 5.96E-06 | 2.89 |
| 7 | 11.88 | Allantoin | 0.000858 | 2.07 |
| 8 | 12.15 | Creatinine | 0.012874 | 0.77 |
| 9 | 12.99 | Pentitol | 0.001807 | 0.67 |
| 10 | 16.54 | Mannose | 0.044428 | 0.40 |
| 11 | 17.09 | Lysine | 0.017295 | 1.29 |
| 12 | 17.16 | Hexadecanoate | 0.024968 | 1.63 |
| 13 | 19.51 | Urate | 0.037078 | 0.43 |
| 14 | 19.99 | Galactose | 0.027866 | 1.26 |
| 15 | 21.23 | Octadecanoate | 0.033645 | 1.30 |
| 16 | 21.33 | Eicosatetraenoate | 0.018562 | 1.56 |
| 17 | 21.99 | Arachidonate | 0.042958 | 1.36 |
| 18 | 22.97 | Inosine | 0.003392 | 0.41 |

Table3. Differentially expressed metabolites in liver to be accountable for the separation between K group and C group

|  |  |  | Average p values of the ANOVA |  |
| --- | --- | --- | --- | --- |
| No. | Retention Time | Metabolites | and Kruskal-Wallis tests | Fold Change |
|  |  |  | K v.s.Control | K v.s.Control |
| 1 | 6.28 | Glycine | 0.039594 | 1.29 |
| 2 | 6.33 | 2-Hydroxybutyrate | 0.001241 | 1.87 |
| 3 | 7.01 | 2-Aminobutyrate | 0.000519 | 1.97 |
| 4 | 7.12 | Urea | 1.17E-05 | 1.66 |
| 5 | 7.64 | Valine | 0.000722 | 1.33 |
| 6 | 8.38 | Ethanolamine | 0.000886 | 1.44 |
| 7 | 8.49 | Glycerol | 0.010937 | 1.21 |
| 8 | 8.75 | Isoleucine | 0.001729 | 1.34 |
| 9 | 8.82 | Prolinee | 0.000113 | 1.44 |
| 10 | 8.94 | Glycine | 0.000492 | 1.29 |
| 11 | 9.65 | Serine | 0.033354 | 1.30 |
| 12 | 13.06 | Alanine | 0.005375 | 1.25 |
| 13 | 11.08 | Aminomalonate | 0.000146 | 1.37 |
| 14 | 11.56 | D-Ribofuranose | 0.020655 | 1.26 |
| 15 | 11.72 | Aspartate | 0.03231 | 1.30 |
| 16 | 11.88 | Allantoin | 1.57E-05 | 2.26 |
| 17 | 12.15 | Cysteine | 0.011729 | 1.52 |
| 18 | 12.71 | Hypotaurine | 0.006966 | 1.65 |
| 19 | 13.06 | Phenylalanine | 0.003397 | 1.43 |
| 20 | 14.05 | Ribitol | 2.52E-05 | 1.38 |
| 21 | 15.41 | Hypoxanthine | 0.000836 | 0.62 |
| 22 | 15.58 | Ornithinee | 0.001132 | 1.46 |
| 23 | 17.09 | Lysine | 9.32E-05 | 1.51 |
| 24 | 17.16 | Hexadecanoate | 0.028124 | 1.53 |
| 25 | 18.52 | Lactobionate | 2.17E-05 | 1.34 |
| 26 | 18.83 | Hexadecanoate | 0.001786 | 1.55 |
| 27 | 19.49 | Octadecadienoate | 0.001773 | 1.93 |
| 28 | 15.92 | Galactose | 5.71E-05 | 1.47 |
| 29 | 21.23 | Octadecanoate | 0.000668 | 1.67 |
| 30 | 21.33 | Eicosatetraenoate | 8.36E-05 | 2.23 |
| 31 | 21.99 | Arachidonate | 0.001907 | 1.90 |
| 32 | 22.93 | Docosahexaenoate | 0.011889 | 1.48 |
| 32 | 22.97 | Inosine | 0.000572 | 0.27 |
| 33 | 23.39 | Cellobiose | 0.004434 | 1.50 |


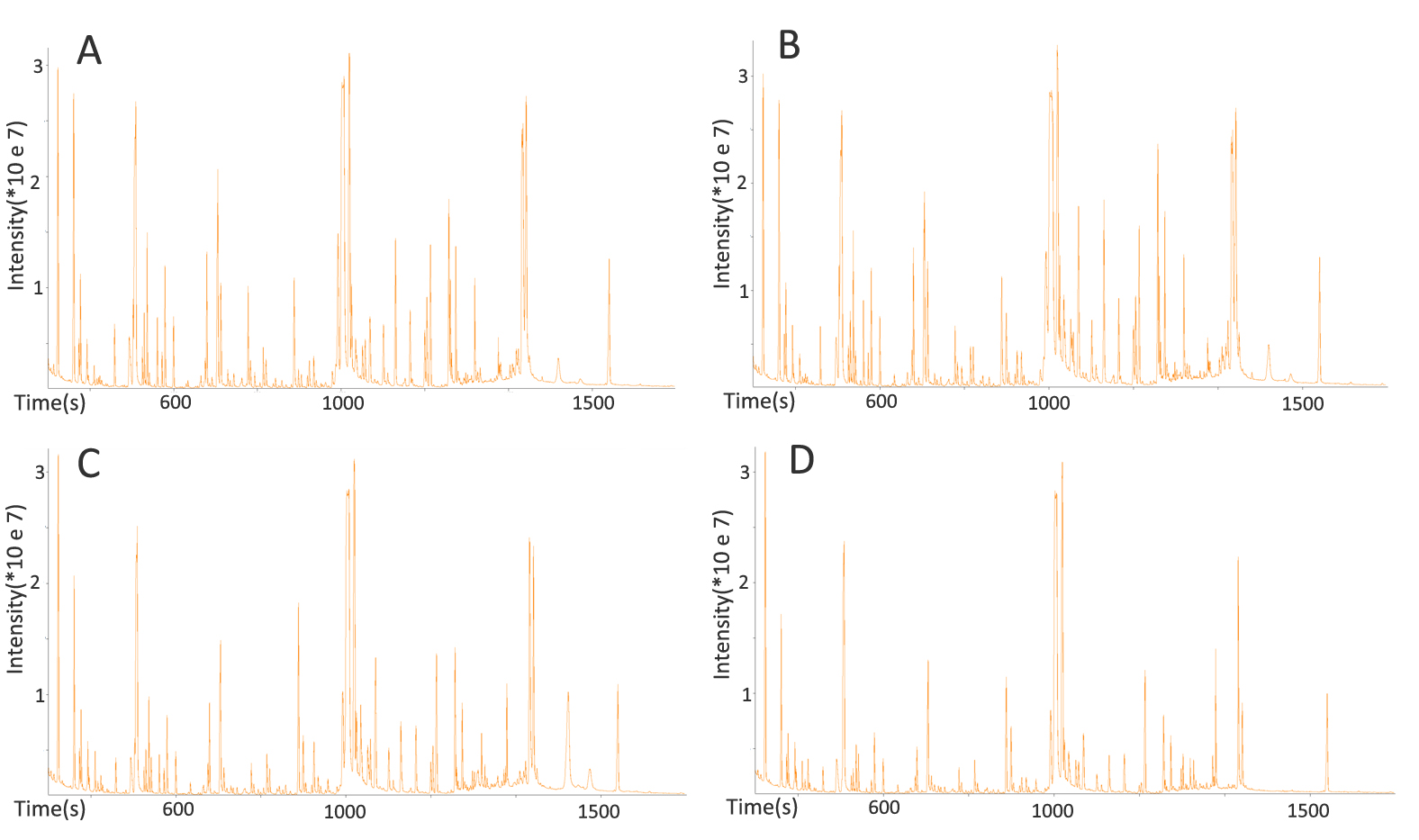


Figure 1. Visualization of biochemical effects of heat stress in rats interfere with kisspeptin-10 using a metabolic profiling approach. Typical GC/MS spectra of liver samples from C (A), H (B), KH (C), K (D) groups. C: control group; H: Heat stress group; KH group: Kisspeptin+Heat stress group; K group: Kisspeptin group.


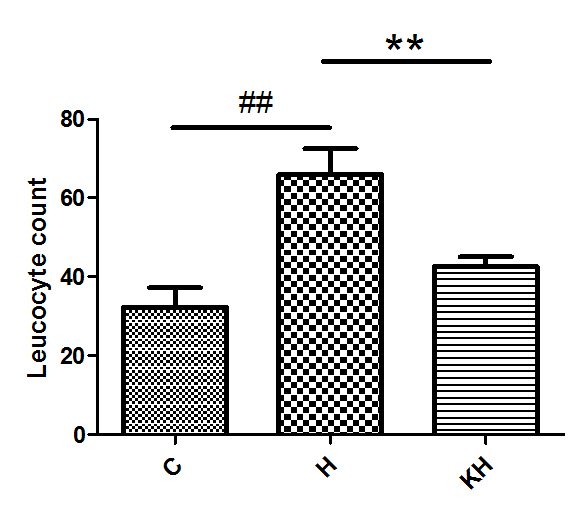


Figure 2. The percentage of the total leucocyte count in liver sections. C: control group; H: Heat stress group; KH group: Kisspeptin+Heat stress group; K group: Kisspeptin group.


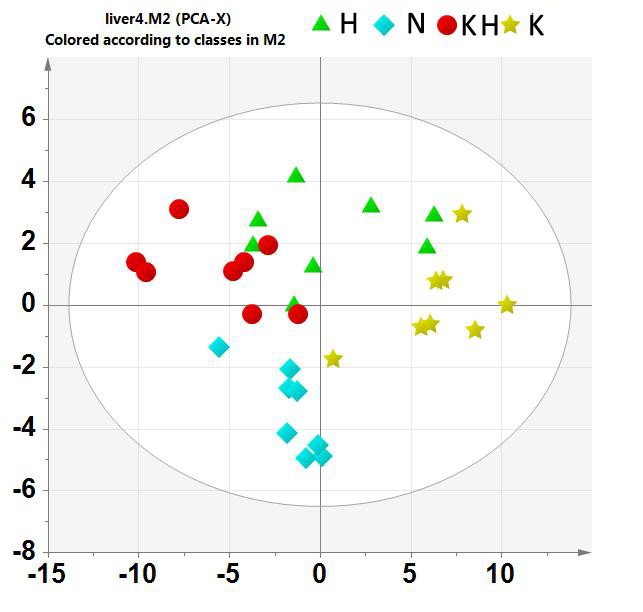


Figure3. PCA scores plot of all samples in different groups. The blue square is the C group, the red dot is the H group, the green [triangle](javascript:void(0);) is the KH group and the yellow asterisks is K group. C: control group; H: Heat stress group; KH group: Kisspeptin+Heat stress group; K: Kisspeptin group.


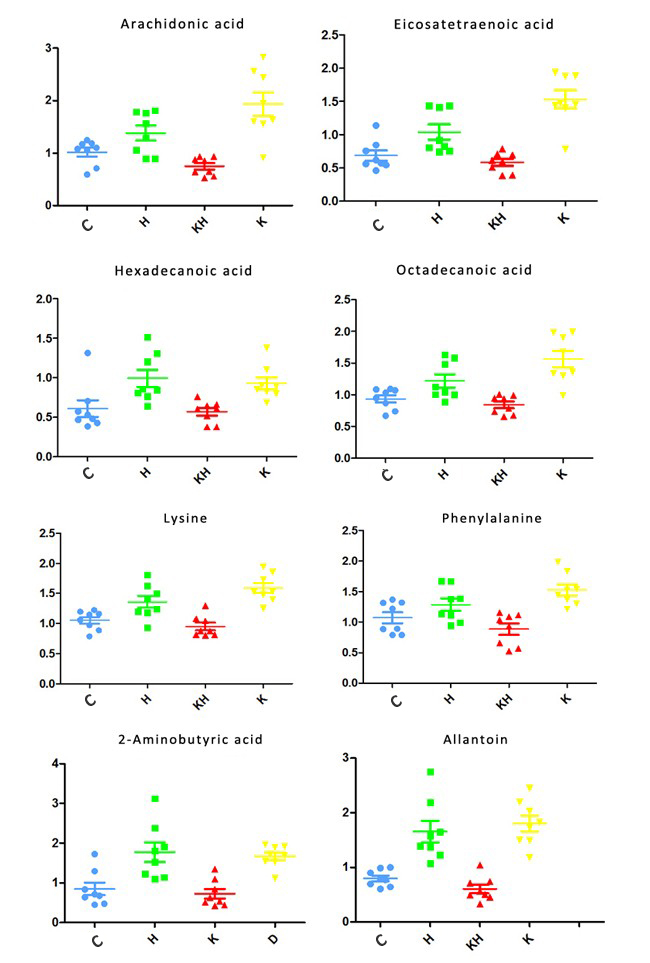


Figure4. Normalized mean peak areas of metabolites in 4 groups.
